# Serosurvey of *Anaplasma phagocytophilum*, *Babesia microti*, and *Ehrlichia chaffeensis* in Quilombola Communities of Southern Brazil

**DOI:** 10.3390/pathogens14040318

**Published:** 2025-03-26

**Authors:** Danilo Alves de França, Louise Bach Kmetiuk, Filipe Pereira da Silva, Giovanni Kalempa Panazzolo, Leandro Meneguelli Biondo, Orlei José Domingues, Giovani Marino Fávero, Ana Íris de Lima Duré, Alexander Welker Biondo

**Affiliations:** 1 Department of Animal Production and Preventive Veterinary Medicine, School of Veterinary Medicine and Animals Science, São Paulo State University, Botucatu 18618-687, Brazil; danilo.franca@unesp.br; 2 Zoonosis Surveillance Unit, City Secretary of Health, Curitiba 81265-320, Brazil; louisebachk@gmail.com; 3 Service of Virology and Rickettsiosis, Octavio Magalhaes Institute, Belo Horizonte 30510-010, Brazil; filipeps.1919@gmail.com (F.P.d.S.); ana.dure@funed.mg.gov.br (A.Í.d.L.D.); 4 Graduate College of Pharmaceutical Sciences, State University of Ponta Grossa, Ponta Grossa 84030-900, Brazilojdsoares@uepg.br (O.J.D.); gmfavero@uepg.br (G.M.F.); 5 Interdisciplinary Graduate Studies, University of British Columbia, Kelowna, BC V6T 1Z4, Canada; leandromet@gmail.com; 6 Department of Veterinary Medicine, Federal University of Paraná, Curitiba 80060-240, Brazil

**Keywords:** hemoparasites, surveillance, tick-borne, public health, One Health

## Abstract

Although *quilombola* individuals and their dogs may be exposed to hemoparasites such as *A. phagocytophilum*, *B. microti*, and *E. chaffeensis*, no study to date has been conducted in these populations. The aim of this study was to investigate the presence of antibodies against *Anaplasma phagocytophilum*, *Babesia microti,* and *Ehrlichia chaffeensis* in humans and dogs from *quilombola* communities in Brazil. Serum samples from humans and dogs were collected from four rural *quilombola* communities and analyzed using indirect immunofluorescence assays. The results revealed antibody levels of 8% for *A. phagocytophilum*, 3% for *B. microti,* and 1% for *E. chaffeensis* in humans and 60%, 50%, and 65%, respectively, in dogs. Notably, women were significantly more likely to be seropositive for *A. phagocytophilum* than men (*p* = 0.0289). Dogs from the Serra do Apon community more commonly had *A. phagocytophilum* (*p* = 0.0477) and *B. microti* (*p* = 0.0448) than those from the other areas. To the best of our knowledge, this is the first study to report human exposure to *A. phagocytophilum* and the ocurrence of *B. microti* in Brazil. The antibody level of vector-borne diseases in humans is a public health concern, particularly in vulnerable populations and rural areas. The dogs were universally hosted *Rhipicephalus sanguineus* ticks, suggesting their possible role in transmission. Thus, further epidemiological surveillance studies should be conducted in vulnerable populations to mitigate the impact of such zoonotic diseases.

## 1. Introduction

*Quilombola* communities, descendants of the former black slaves in Brazil, face public health challenges, including limited access to living areas, lack of specific public policies, and exposure to zoonotic diseases [1]. These communities typically reside in rural, semi-isolated areas where they rely on backyard livestock and agriculture, which brings them into close contact with domestic and wild animals [2,3]. This lifestyle may increases their exposure to zoonotic agents, including vector-borne hemoparasites, which are often associated with tick infestations and are common in tropical developing countries [4].

Hemoparasites are mainly transmitted through hematophagous vectors, such as ticks [5]. In Brazil, domestic animals, particularly in economically vulnerable regions, are frequently infested with ticks [4]. *Anaplasma platys*, *Babesia canis*, and *Ehrlichia canis* are frequently reported in dogs worldwide and are commonly transmitted by the tick *Rhipicephalus sanguineus* [6]. In cattle and horses, infections with *Anaplasma marginale*, *Babesia bovis*, and *Babesia caballi*, transmitted by the ticks *Rhipicephalus microplus*, *Amblyomma cajennense*, and *Dermacentor nitens*, are more common [7,8,9]. Although *Anaplasma phagocytophilum*, *Babesia microti*, and *Ehrlichia chaffeensis* have been reported in humans worldwide, they have not yet been detected in Brazil [10,11,12].

*A. phagocytophilum* is an obligate intracellular pathogen that infects granulocytes and causes human granulocytic anaplasmosis [13]. The disease is transmitted by Ixodes ticks and is associated with fever, headache, myalgia, arthralgia, leukopenia, thrombocytopenia, and elevated liver enzymes [10]. In Brazil, studies have reported infections in dogs, horses, *R. sanguineus*, and *A. cajennense* in different regions of the country, suggesting a zoonotic risk to humans [14,15].

*B. microti*, another obligate intracellular pathogen, causes babesiosis in humans. This pathogen is also transmitted by Ixodes ticks and is characterized by symptoms such as fever, hemolytic anemia, thrombocytopenia, sweating, anorexia, acute respiratory failure, and renal failure [12]. In Brazil, no study has detected cases of *B. microti* in humans or animals, except for a single report of clinical manifestations in a Polish traveler shortly after his arrival in Brazil [16].

*E. chaffeensis* is an obligate intracellular pathogen that infects monocytes and causes human monocytic ehrlichiosis. This pathogen is transmitted by *Amblyomma* ticks and causes fever, headache, myalgia, vomiting, thrombocytopenia, respiratory failure, and disseminated intravascular coagulation [11]. In Brazil, *E. chaffeensis* infections and exposure have been extensively reported [17], including in marsh deer (*Blastocerus dichotomus*), carthorses, dogs, cats, and healthy humans [18]. Additionally, nine human cases of ehrlichiosis were recorded in Brazil, associated with fever, endocarditis, encephalitis, and a history of tick bites [19].

Although *quilombola* communities and their dogs may be exposed to hemoparasites such as *A. phagocytophilum*, *B. microti*, and *E. chaffeensis*, to the best of our knowledge, no studies have investigated these populations specifically. Therefore, we aimed to investigate the presence of antibodies against *A. phagocytophilum*, *B. microti*, and *E. chaffeensis* in *quilombola* communities and their dogs.

## 2. Materials and Methods

### 2.1. Ethical Statement

This study was approved by the Ethics Committee on Human Health of the Brazilian Ministry of Health (protocol: 53828121.1.0000.0105) approved on 29 November 2021 and the Ethics Committee for the Use of Animals of the State University of Ponta Grossa (protocol: 22.000075139-9).

### 2.2. Study Area

This study was conducted in four *quilombola* communities—Limitão, Mamans, Serra do Apon, and Tronco—located in the rural regions of southern Brazil. These communities, known as “quilombos”, were established at the end of the 19th century by individuals fleeing slavery. As a form of subsistence, members dedicate themselves to the cultivation of plants and livestock [20]. *Quilombola* settlements are typically situated approximately 60 km (37 miles) from main roads, accessible only by unmaintained gravel and sand roads. The study area comprises natural and degraded regions of the Atlantic Forest and Cerrado biomes, characterized by a humid temperate climate, an average temperature of 17.5 °C, and an average rainfall of 1495 mm^3^.

### 2.3. Sample Size

The minimum sample size for logistic regression was calculated based on an estimated seroprevalence of 50% in the *quilombola* population, as no prevalence data were available for the state. The calculation assumed a 0.05 probability of rejecting the null hypothesis (type I error), 20% probability of failing to reject the null hypothesis under the alternative hypothesis (type II error), and a minimum detectable odds ratio (OR) of 1.5. The calculations based on the available “Sample Size Calculators for designing clinical research” resulted in a minimum of 194 individuals [21]. A total of 200 human blood samples were collected from the studied communities, with the following distribution: 45 samples from Limitão, 42 from Mamans, 74 from Serra do Apon, and 39 from Tronco. Additionally, blood samples were collected from 20 dogs in these communities, despite challenges associated with physical restraint.

### 2.4. Sample Collection

Sample collection was conducted in collaboration with the Association of Rural *Quilombola* Communities of Castro County, an organization dedicated to strengthening community ties, preserving African cultural heritage, and advocating for public policies. Certified nurses, pharmacists, veterinarians, and biologists contributed to this study.

We conducted six field visits between December 2021 and March 2022. All adult participants provided informed consent and answered an epidemiological questionnaire before the samples were collected. For illiterate individuals, the consent form was explained in advance, and their fingerprint was used in place of a signature. For children aged < 18 years, parental or legal guardian consent was required. For dogs, their owners signed a formal consent form before the samples were taken.

Blood samples were collected using appropriate techniques: 10 mL of blood was drawn from human participants via cephalic venipuncture by certified nurses and, from dogs, via jugular venipuncture by qualified veterinarians after physical restraint. All blood samples were stored in tubes without anticoagulants and centrifuged at 1500 rpm for 5 min. The serum was then separated and stored at −80 °C until laboratory processing. All the dogs were vaccinated, dewormed, and administered flea medication. At the time of blood collection, all dogs had ticks and/or fleas; however, these ectoparasites were not collected for further analysis.

### 2.5. A. phagocytophilum Antibody Detection

Serum samples were tested for *A. phagocytophilum* antibodies using an indirect immunofluorescence assay (IFA) immunoglobulin G (IgG) Kit (Fuller Laboratories, Fullerton, CA, USA), according to the manufacturer’s protocol. The kit included both positive and negative controls. For human samples, reactions were performed using conjugated anti-human IgG antibodies (Bethyl Laboratories, Montgomery, TX, USA). For canine samples, canine anti-IgG conjugated antibodies were used (Zoonosis Control Center, São Paulo, Brazil). Samples with antibody titers exceeding the kit’s cut-off value of 1:80 were classified as seropositive.

### 2.6. B. microti Antibody Detection

Serum samples were tested for *B. microti* antibodies using an IFA IgG Kit (Fuller Laboratories, Fullerton, CA, USA), according to the manufacturer’s protocol. The kit contained both positive and negative controls. Conjugated anti-human IgG antibodies (Bethyl Laboratories, Montgomery, TX, USA) were used for the reactions with human samples. Canine anti-IgG-conjugated antibodies were used for the animal samples (Zoonosis Control Center, São Paulo, Brazil). Samples with antibody titers exceeding the kit’s cutoff value of 1:64 were classified as seropositive.

### 2.7. E. chaffeensis Antibody Detection

Serum samples were tested using an indirect immunofluorescence assay with an *E. chaffeensis* IFA IgG Kit (Fuller Laboratories Fullerton, CA, USA), according to the manufacturer’s protocol. The kit contained both positive and negative controls. For human samples, reactions were performed using conjugated anti-human IgG antibodies (Bethyl Laboratories, Montgomery, TX, USA). For canine samples, canine anti-IgG conjugated antibodies were used (Zoonosis Control Center, São Paulo, Brazil). Samples with antibody titers exceeding the kit’s cutoff value of 1:40 were classified as seropositive.

### 2.8. Data Analysis

Univariate statistical analysis was conducted using the chi-square test to assess the associations between seropositivity and categorical variables. The analysis focused on assessing potential correlations between infection status in humans and animals and factors such as community of origin, age, sex, education level, access to forest areas, history of flea and tick bites, contact with dogs, exposure to canine abortions, and history of tick control. Statistical significance was set at *p* < 0.05. All statistical analyses were performed using SAS Studio (version 3.81, SAS Institute Inc., Cary, NC, USA).

## 3. Results

**Figure 1:**
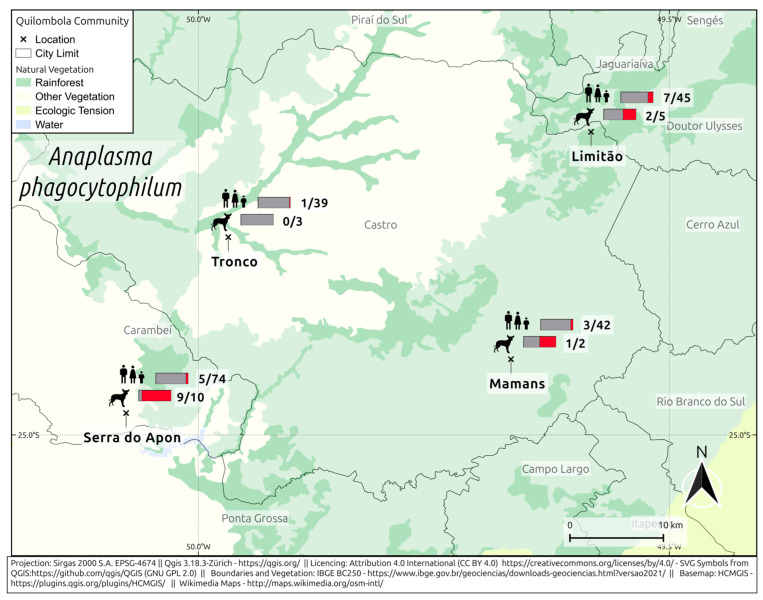
Frequency of anti-*A. phagocytophilum* in *quilombola* individuals and their dogs from southern Brazil.

A total of 16/200 humans (8%; 95% CI: 4.98–12.60) and 12/20 dogs (60%; 95% CI: 38.66–78.12) were seropositive for anti-*A. phagocytophilum* (Figure 1). In the Limitão community, 16% (7/45) of humans and 40% (2/5) of dogs were positive. In the Mamans community, 7% (3/42) of humans and 50% (1/2) of dogs tested positive for the virus. In Serra do Apon, 7% (5/74) of humans and 90% (9/10) of dogs were seropositive. In the Tronco community, 3% (1/39) of humans were seropositive and all dogs were seronegative (0/3).

**Figure 2:**
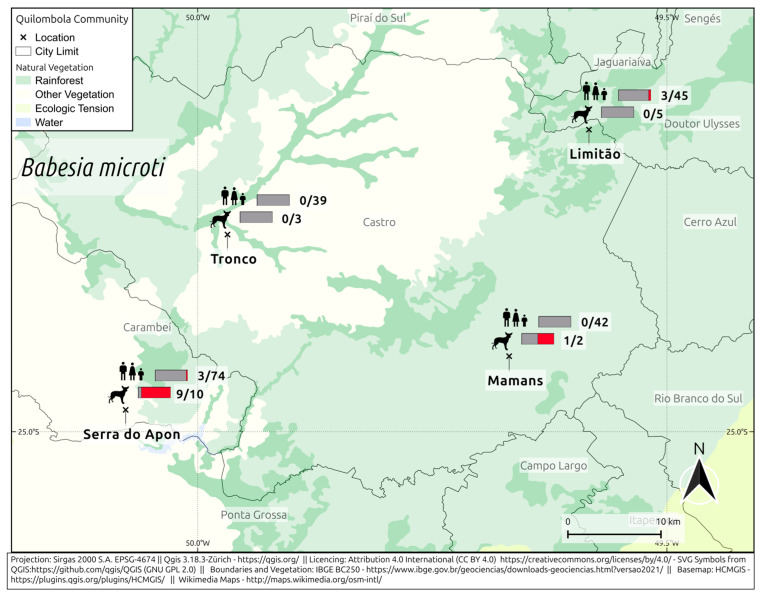
Frequency of anti- *B. microti* in *quilombola* individuals and their dogs from southern Brazil.

For antibodies against *B. microti*, 6/200 humans (3%; 95% CI: 1.38–6.39) and 10/20 dogs (50%; 95% CI: 29.93–70.07) were seropositive (Figure 2). In the Limitão community, 7% (3/45) of humans were seropositive, and all dogs were seronegative. In Mamans, 50% (1/2) of dogs were positive, and all humans were seronegative. In Serra do Apon, 4% (3/74) of humans and 90% (9/10) of dogs were seropositive. In Tronco, all humans and dogs were seronegative.

**Figure 3:**
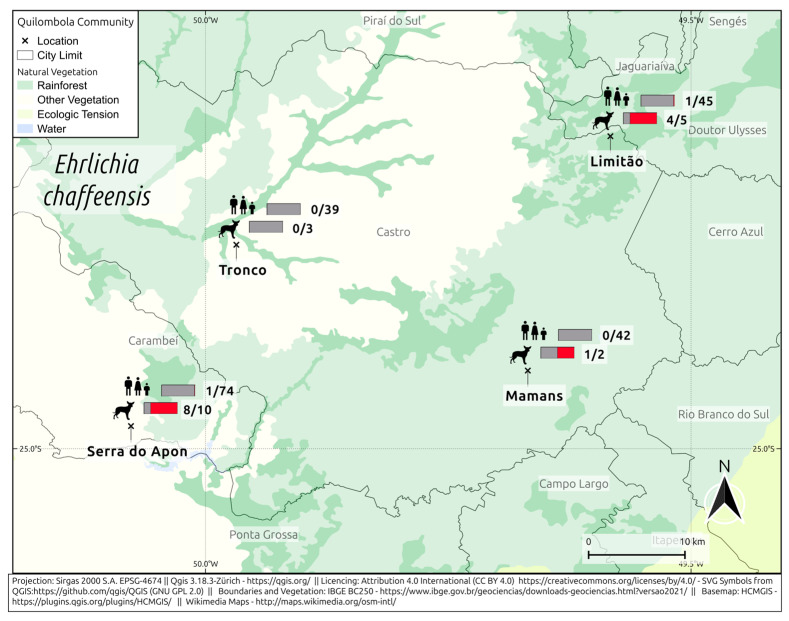
Frequency of anti-*E. chaffeensis* in *quilombola* individuals and their dogs from southern Brazil.

For antibodies against *E. chaffeensis*, 2/200 humans (1%; 95% CI: 0.27–3.57) and 13/20 dogs (65%; 95% CI: 43.29–81.88) were seropositive (Figure 3). In the Limitão community, 2% (1/45) of the humans and 80% (4/5) of the dogs were seropositive. In Mamans, 50% (1/2) of dogs were seropositive, and all humans were seronegative. In Serra do Apon, 1% (1/74) of humans, and 80% (8/10) of dogs were seropositive. In Tronco, all humans and dogs were seronegative.

There was one case of human coexposure to *A. phagocytophilum* and *B. microti* and one case to *A. phagocytophilum* and *E. chaffeensis*. Canine coexposure to different combinations of pathogens was observed: *A. phagocytophilum* and *B. microti* in eight dogs, *A. phagocytophilum* and *E. chaffeensis* in eight dogs, and *B. microti* and *E. chaffeensis* in seven dogs. Moreover, triple exposures to pathogens were recorded in five dogs.

Table 1, Table 2, Table 3, Table 4 and Table 5 present the results of the statistical analyses, highlighting the main epidemiological variables related to hemoparasite infection and their respective ORs. Owing to the low seropositivity observed for *E. chaffeensis* in humans, an association analysis could not be performed.

A statistically significant association was observed between *A. phagocytophilum* seropositivity and sex (*p* = 0.0289), with women being 5.38 times more likely to test positive than men. No statistically significant associations were found for the other analyzed variables. Although no direct association was observed between seropositivity and a history of tick bites, the high prevalence of tick exposure among *quilombolas* may have obscured potential differences.

Although the ORs indicated that individuals from Limitão and Serra do Apon as well as those with a history of tick bites and contact with dogs were more likely to be seropositive, these associations were not statistically significant. Moreover, no significant associations were found between *B. microti* positivity and any of the analyzed epidemiological variables. Notably, one individual from Limitão reported experiencing fever following a tick bite.

A significant association was observed between *A. phagocytophilum* seropositivity and community of origin (*p* = 0.0477). The likelihood of seropositivity was highest in Serra do Apon, where individuals were 9, 13.5, and 44.3 times more likely to be seropositive compared with those from Mamans, Limitão, and Tronco, respectively.

Similarly, *B. microti* seropositivity was significantly associated with community of origin (*p* = 0.0448). Individuals in Serra do Apon were 9, 69.6, and 44.3 times more likely to be seropositive compared with those in Mamans, Limitão, and Tronco, respectively. Although the ORs indicate a higher probability of exposure in the Serra do Apon than in the other communities, the wide confidence intervals for some comparisons indicate the need for cautious interpretation. Furthermore, there was no association between *E. chaffeensis* seropositivity and the variables evaluated.

## 4. Discussion

This is the first study on the human seropositivity for these hemoparasites in Brazil. The prevalences of 8% (16/200) for *A. phagocytophilum* and 3% (6/200) for *B. microti* highlight an emerging public health concern in these *quilombola* communities, with potential implications for other regions in Brazil. The high seropositivity rates observed in dogs for the three parasites investigated and the detection of antibodies against the same pathogens in their owners suggest that these animals may play an important role as sources of infection. Furthermore, it is crucial to investigate the impact of these infections in dogs, considering the risk of misdiagnosing tick-borne diseases. Misattributing the cause to other species within the same genus can lead to inappropriate treatments, which may worsen the condition or delay effective management [5,22,23].

All dogs sampled in this study were infested with *R. sanguineus* ticks, which are known vectors of hemoparasites [24,25,26,27]. Despite this observation, a notable limitation of this study was the absence of tick collection and molecular analysis, which could have provided more detailed insights into the role of ticks in transmission. The high seropositivity for the pathogens in dogs strongly suggests that these ectoparasites are the main vectors of these pathogens in the canine population. Furthermore, considering the close contact between dogs and their owners, it is plausible that *R. sanguineus* ticks could also play a role in transmitting infections to humans, especially since most of the owners reported a history of tick bites.

Despite their widespread use, IFAs have limitations, including the potential for false-negative results. Factors such as low antibody titers in the early stages of infection, individual variations in the immune response, and cross-reactivity with other pathogens may affect sensitivity. IFAs may fail to detect some seropositive cases, particularly in immunocompromised individuals or when antibody levels decline over time. These limitations highlight the need for complementary diagnostic approaches to improve the detection rates [28].

Although dogs and humans share the same source of infection, ticks, the results of this study indicate a higher prevalence of *A. phagocytophilum* infection in humans than other hemoparasites. This discrepancy raises questions about the possible additional factors that may influence *A. phagocytophilum* infection in humans, including from occupational exposure, contact with blood or tissues from infected animals, and blood transfusions [29]. The higher susceptibility of humans to *A. phagocytophilum* infection could also be related to immunological or genetic factors that modulate the response to the pathogen [29]. Previous studies on *A. phagocytophilum* in dogs and *R. sanguineus* ticks in Brazil support the hypothesis that ticks act as vectors in these communities [14]. Moreover, these rural communities are close to livestock such as horses, which are associated with *A. phagocytophilum* infections in Brazil [15].

To the best of our knowledge, this is the first study to report the occurrence of *B. microti* in both humans and animals in Brazil, representing a significant advancement in understanding the epidemiology of this hemoparasite in the country. To date, the only report involving *B. microti* in Brazil was that of a Polish traveler who developed babesiosis upon returning to Poland after visiting Brazil, although it was not confirmed whether the infection occurred in Brazil [16]. The findings of this study confirm the presence of this pathogen in Brazil and emphasize the need for epidemiological surveillance of babesiosis in tropical regions. Given that the tick species in these areas differ from those in temperate regions, they may influence disease transmission dynamics [30,31].

Furthermore, statistical analysis revealed that women were more likely to test seropositive for *A. phagocytophilum* than men. This could be because the women had more contact with dogs and ticks, making them more susceptible to infection. In the Serra do Apon community, the dogs showed significantly higher seropositivity than those in other communities, suggesting a higher tick infestation rate in this area. Consequently, focused investigations on tick populations in this region, as well as intensive de-ticking efforts, are essential to prevent further infections.

Infectious diseases have long affected vulnerable populations. The *quilombola* communities in Brazil, which are an important part of the country’s history owing to their origins in the aftermath of slavery, continue to face the consequences of limited access to basic healthcare, adequate housing, and sanitation [32,33,34]. These challenges also contribute to the lack of dog population control and deworming in *quilombola* communities. Similarly, other vulnerable populations in Brazil have shown higher rates of zoonotic infections, highlighting the need to strengthen animal health within governmental health initiatives and assess the situation from a One Health perspective [35].

Furthermore, the non-specific nature of the symptoms of these diseases, such as fever, myalgia, and headache, often leads to misdiagnoses, with symptoms being attributed to more common conditions like dengue, malaria, or chikungunya [36]. This situation is aggravated by the lack of training for healthcare professionals in recognizing emerging zoonotic diseases [36]. This highlights the urgent need to expand the training of health systems and implement epidemiological surveillance programs that include specific tests for hemoparasites, especially in at-risk populations [37].

## 5. Conclusions

To the best of our knowledge, this is the first study to report human exposure to *A. phagocytophilum* and the occurrence of *B. microti* infections in Brazil. The observed antibody levels for vector-borne diseases in humans represent a substantial public health concern, particularly in vulnerable populations and rural areas. The dogs in this study were universally infested with *R. sanguineus* ticks, suggesting their possible role in transmission. Therefore, additional epidemiological surveillance studies should be conducted on vulnerable populations to better understand and reduce the impact of zoonotic diseases.

## Figures and Tables

**Table 1:** Analysis of epidemiological variables associated with seropositivity for *Anaplasma phagocytophilum* in the Brazilian *quilombola* population.

Variable	Sample Size	No. Seropositive (%)	OR (95% CI)	*p* Value
*Quilombola* community				0.1541
Limitão	45	7 (16%)	1.0 (ref)	
Mamans	42	3 (7%)	2.39 (0.57–9.95)	
Serra do Apon	74	5 (7%)	2.54 (0.75–8.55)	
Tronco	39	1 (3%)	7.00 (0.82–59.6)	
Age				0.3881
Young (1 to 18)	44	4 (9%)	1.0 (ref)	
Adults (19 to 59)	127	8 (6%)	1.48 (0.42–5.20)	
Seniors (≥60)	29	4 (14%)	0.62 (0.14–2.72)	
Sex				0.0289
Female	118	14 (12%)	1.0 (ref)	
Male	82	2 (2%)	5.38 (1.18–24.3)	
Education				0.4022
Illiterate	36	2 (6%)	1.0 (ref)	
Elementary school	133	13 (10%)	0.54 (0.11–2.52)	
High school	31	1 (3%)	1.76 (0.15–20.5)	
Access to forest areas				0.6624
Yes	170	13 (8%)	1.0 (ref)	
No	30	3 (10%)	0.74 (0.19–2.79)	
Flea bites				0.9371
Yes	161	13 (8%)	1.0 (ref)	
No	39	3 (8%)	1.05 (0.28–3.89)	
Tick bites				0.9094
Yes	140	11 (8%)	1.0 (ref)	
No	60	5 (8%)	0.93 (0.31–2.82)	
Dog contact				0.9094
Yes	177	13 (7%)	1.0 (ref)	
No	23	3 (13%)	0.93 (0.31–2.82)	
Dog abortion contact				
Yes	38	3 (8%)	1.0 (ref)	0.3503
No	162	13 (8%)	0.52 (0.13–2.01)	

*p* value < 0.05 indicates statistical difference within categories; 1.0 (ref.): reference category; OR (95% CI): odds ratio (95% confidence interval).

**Table 2:** Analysis of epidemiological variables associated with seropositivity for *Babesia microti* in the Brazilian *quilombola* population.

Variable	Sample Size	No. Seropositive (%)	OR (95% CI)	*p* Value
*Quilombola* community				0.2026
Limitão	45	3 (7%)	1.0 (ref)	
Mamans	42	0 (0%)	7.00 (0.35–139)	
Serra do Apon	74	3 (4%)	1.69 (0.32–8.76)	
Tronco	39	0 (0%)	6.50 (0.32–129)	
Age				0. 2514
Young (1 to 18)	44	2 (5%)	1.0 (ref)	
Adults (19 to 59)	127	2 (2%)	2.97 (0.40–21.8)	
Seniors (≥60)	29	2 (7%)	0.64 (0.08–4.84)	
Sex				0.6508
Female	118	3 (3%)	1.0 (ref)	
Male	82	3 (4%)	0.68 (0.13–3.49)	
Education				0.4442
Illiterate	36	0 (0%)	1.0 (ref)	
Elementary school	133	5 (4%)	3.12 (0.16–57.8)	
High school	31	1 (3%)	1.17 (0.13–10.4)	
Access to forest areas				0.5524
Yes	170	6 (4%)	1.0 (ref)	
No	30	0 (0%)	2.41 (0.13–43.9)	
Flea bites				0.8125
Yes	161	5 (3%)	1.0 (ref)	
No	39	1 (3%)	1.30 (0.14–11.5)	
Tick bites				0.4799
Yes	140	5 (4%)	1.0 (ref)	
No	60	1 (2%)	2.18 (0.24–19.1)	
Dog contact				0.6972
Yes	177	6 (3%)	1.0 (ref)	
No	23	0 (0%)	1.78 (0.09–32.7)	
Dog abortion contact				0.8825
Yes	38	1 (3%)	1.0 (ref)	
No	162	5 (3%)	0.84 (0.09–7.48)	

*p* value < 0.05 indicates statistical difference within the categories; 1.0 (ref.): reference category; OR (95% CI): odds ratio (95% confidence interval).

**Table 3:** Analysis of epidemiological variables associated with seropositivity for *Anaplasma phagocytophilum* in the *quilombola* dogs.

Variable	Sample Size	No. Seropositive (%)	OR (95% CI)	*p* Value
*Quilombola* community				0.0477
Serra do Apon	10	9 (90%)	1.0 (ref)	
Mamans	2	1 (50%	9.00 (0.28–285)	
Limitão	5	2 (40%)	13.5 (0.87–207)	
Tronco	3	0 (0%)	44.3 (1.43–1365)	
Access to forest areas				0.1532
Yes	9	7 (78%)	1.0 (ref)	
No	11	5 (45%)	4.20 (0.58–30.0)	
Has tick control				0.7988
Yes	3	2 (67%)	1.0 (ref)	
No	17	10 (83%)	0.71 (0.05–9.49)	

*p* value < 0.05 indicates statistical difference within the categories; 1.0 (ref.): reference category; OR (95% CI): odds ratio (95% confidence interval).

**Table 4:** Analysis of epidemiological variables associated with seropositivity for *Babesia microti* in the *quilombola* dogs.

Variable	Sample Size	No. Seropositive (%)	OR (95% CI)	*p* Value
*Quilombola* community				0.0448
Serra do Apon	10	9 (90%)	1.0 (ref)	
Mamans	2	1 (50%)	9.00 (0.28–285)	
Limitão	5	0 (0%)	69.6 (2.39–2022)	
Tronco	3	0 (0%)	44.3 (1.43–1365)	
Access to forest areas				0.6537
Yes	9	4 (44%)	1.0 (ref)	
No	11	6 (55%)	0.66 (0.11–3.92)	
Has tick control				0.5383
Yes	3	2 (67%)	1.0 (ref)	
No	17	8 (47%	2.25 (0.17–29.8)	

*p* value < 0.05 indicates statistical difference within the categories; 1.0 (ref.): reference category; OR (95% CI): odds ratio (95% confidence interval).

**Table 5:** Analysis of epidemiological variables associated with seropositivity for *Ehrlichia chaffeensis* in the *quilombola* dogs.

Variables	Sample Size	No. Seropositive (%)	OR (95% CI)	*p* Value
*Quilombola* community				0.3891
Serra do Apon	10	8 (80%)	1.0 (ref)	
Mamans	2	1 (50%)	4.00 (0.16–95.7)	
Limitão	5	4 (80%)	1.00 (0.06–14.6)	
Tronco	3	0 (0%)	23.8 (0.89–633)	
Access forest areas				0.4266
Yes	9	5 (56%)	1.0 (ref)	
No	11	8 (73%)	0.46 (0.07–3.03)	
Has tick control				0.9477
Yes	3	2 (67%)	1.0 (ref)	
No	17	11 (65%)	1.09 (0.08–14.6)	

*p* value < 0.05 indicates statistical difference within the categories; 1.0 (ref.): reference category; OR (95% CI): odds ratio (95% confidence interval).

## Data Availability

All data used in the present study are included in this manuscript, with no mention of the origin of sensitive data.
